# The Clinical Characteristics and Pathological Patterns of Postinfectious Glomerulonephritis in HIV-Infected Patients

**DOI:** 10.1371/journal.pone.0108398

**Published:** 2014-10-01

**Authors:** Christine A. Murakami, Doaa Attia, Naima Carter-Monroe, Gregory M. Lucas, Michelle M. Estrella, Derek M. Fine, Mohamed G. Atta

**Affiliations:** 1 Department of Medicine, Johns Hopkins University School of Medicine, Baltimore, Maryland, United States of America; 2 Faculty of Medicine, Alexandria, Egypt; 3 Department of Pathology, Johns Hopkins University School of Medicine, Baltimore, Maryland, United States of America; University of São Paulo School of Medicine, Brazil

## Abstract

**Background:**

Postinfectious glomerulonephritis (PIGN), a form of immune complex GN, is not well-defined in HIV-infected patients. This study characterizes PIGN in this patients’ population and determine the impact of histopathological patterns on renal outcome and mortality.

**Methods:**

HIV-infected patients with PIGN from September 1998 to July 2013 were identified. Archived slides were reviewed by a blinded renal pathologist, classified into acute, persistent and healed PIGN. Groups were compared using Wilcoxon rank-sum and Fisher’s exact test. Survival analyses were performed to determine association of histopathological pattern with renal outcome and mortality.

**Results:**

Seventy-two HIV-infected predominantly African American males were identified with PIGN. Median (interquartile range) age and creatinine at the time of renal biopsy was 48 years (41, 53) and 2.5 mg/dl (1.5, 4.9) respectively. Only 2 (3%) had acute PIGN, 42 (58%) had persistent PIGN and 28 (39%) had healed PIGN. Three patients (4%) had IgA-dominant PIGN. Only 46% of the patients had confirmed positive cultures with *Staphylococcus* the most common infectious agent. During a median follow up of 17 months, the pathological pattern had no impact on renal outcome (*P* = 0.95). Overall mortality was high occurring in 14 patients (19%); patients with healed PIGN had significantly increased mortality (*P* = 0.05).

**Conclusion:**

In HIV-infected patients, *Staphylococcus* is the most common cause of PIGN. Renal outcome was not influenced by the histopathological pattern but those with healed PIGN had greater mortality which was potentially due to a confounder not accounted for in the study.

## Introduction

Renal diseases in patients infected with HIV cover a wide array of renal pathologies [1]–[3]. These pathologies could be the direct effect of the HIV-1 virus, such as HIV-1 associated nephropathy (HIVAN), or the consequence of coexisting conditions such as diabetes, hypertension, intravenous drug use, or exposure to antiretroviral medications [4]. The incidence of HIVAN, the most aggressive histologic lesion in the HIV population, has substantially declined in recent years due to the introduction of highly-active antiretroviral therapy (HAART) [5], [6]. However, HIV-immune complex-mediated kidney disease (HIVICK) continues to be a pervasive histologic finding [7]. HIVICK refers to a spectrum of pathological entities consisting of post-infectious glomerulonephritis (PIGN), “lupus-like’ glomerulonephritis, IgA nephropathy, membranoproliferative glomerulonephritis (MPGN), and membranous nephropathy [4]. In a study by Foy *et al*, PIGN was identified as the most common immune-complex glomerulonephritis in HIV patients [4].

In recent years, the disease pattern and epidemiology of PIGN has markedly evolved [8], [9]. Infections with *Staphylococcal* and gram-negative bacteria have now been identified as increasingly common antecedent of PIGN. Studies have also reported a more unfavorable prognosis than two to three decades ago; this has been attributed to changes in the disease profile as well as delayed diagnosis and treatment [8], [10]. Complete remission has been reported in some studies to occur in 26% to 69% of adults with PIGN [8], [11], [12]. While there have been several studies describing the clinicopathologic spectrum of PIGN in the adult population, data on the prevalence and prognosis of PIGN among adults HIV patients are lacking [8], [10], [13]. Histologically, PIGN is characterized by diffuse endocapillary proliferative and exudative glomerulonephritis on light microscopy with granular deposits of complement 3 (C3) and immunoglobulin G (IgG) in the mesangium and glomerular basement membranes on immunofluorescence (IF). On electron microscopy, large subepithelial electron-dense deposits or “humps” are characteristic findings [14]. Although, classic cases of PIGN are diagnosed histologically without difficulty, a large number of patients have PIGN with atypical histological features [10]. In 2003, Haas reviewed 1012 renal biopsies and classified PIGN as acute or subacute glomerulonephritis (GN), persistent or progressive glomerulonephritis and healed or latent glomerulonephritis [15]. In this classification, acute or subacute GN pertains to cases with diffuse mesangial and endocapillary hypercellularity on light microscopy, multiple subepithelial humps on electron microscopy (EM), characteristic granular glomerular basement membrane staining for IgG and or C3 on IF, and clinical findings consistent with acute or subacute kidney injury. Persistent or progressive GN lacks the intense degree of diffuse mesangial and endocapillary hypercellularity seen in acute PIGN, and has variable granular mesangial C3 and or IgG deposits with some capillary wall deposits. On EM, patients with persistent PIGN have fewer subepithelial humps compared to acute PIGN. Healed or latent GN refers to cases with mild mesangial hypercellularity and immunofluorescence (IF) similar to persistent GN, with partially or markedly resorbed subepithelial deposits on EM. Although this classification may be helpful, it is not known whether it has clinical implications, particularly in HIV-infected patients.

In this study, the clinical features of PIGN in HIV-infected patients and their correlation with the different aforementioned pathological patterns are explored. In addition, the impact of these pathological findings on renal outcome and mortality is determined.

## Materials and Methods

### Study Design and Patient Selection

**Figure 1:**
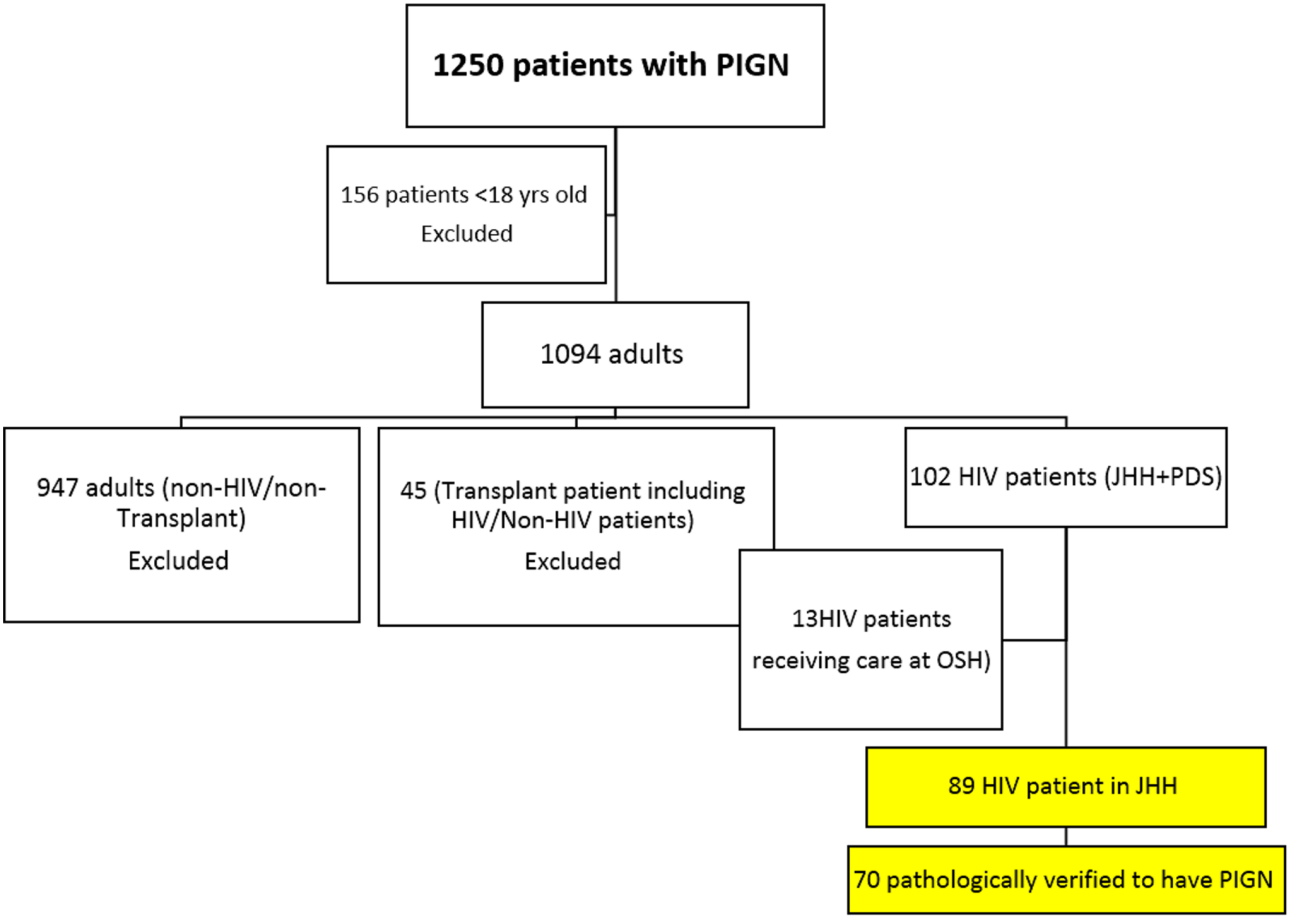
Screening of patients: Out of 1250 patients screened, 72 HIV infected individuals with Post infectious glomerulonephritis were included in the final analysis.

Patients with the diagnosis of PIGN on renal biopsy from September 1998 to July 2013 were identified through linkage with the institutional pathology database. Patients who met all the following criteria were included: 18 years of age and older, diagnosis of HIV-1 infection, hospital admission at Johns Hopkins University from September 1998 to July 2013. Pediatric patients, patients who received any type of organ transplantation as well as those who received care at other institutions were excluded. We initially identified 90 adult HIV-infected patients who met these criteria (Figure 1) but only 72 patients were eventually included in the study due to uncertain pathological diagnoses in 18 cases. The Institutional Review Board of Johns Hopkins University approved the study. Patient records and information were de-identified prior to analysis.

### Data Collection and Definitions

The diagnosis of PIGN was established from the pathological report generated by the renal pathologist at the time of the renal biopsy. A renal pathologist, who was blinded to clinical characteristics, reviewed the slides of the 90 patients screened for this study. All renal biopsies were processed by light microscopy and EM. All but 4 slides were also examined by IF. The patients with PIGN were further subclassified into acute, persistent, or healed PIGN based on the system proposed by Haas *et al* summarized in Table 1 [15]. Demographic, clinical, laboratory and pharmaceutical data collected at the time of renal biopsy and at 2 months before and after the biopsy, were abstracted from electronic patient records. The following clinical definitions were used: HIV-1 infection based on documented diagnosis from medical records and or use of antiretroviral medications; diabetes and hypertension were defined by prescription of anti-diabetic or anti-hypertensive medications, respectively; hepatitis C virus (HCV) infection was based on presence of hepatitis C viral antibodies and/or detection of hepatitis C viral load by polymerase chain reaction. Cirrhosis was based on patient medical history and/or liver biopsy findings and end-stage renal disease (ESRD) was defined as initiation of renal replacement therapy. Nephrotic-range proteinuria was defined by >3.0 g/d (if 24 hour urine collected) or >3.0 g/g creatinine (by random urine protein-to-creatinine ratio).

**Table 1:** Histopathological classification of postinfectious glomerulonephritis.

	Acute PIGN	Persistent PIGN	Healed PIGN
Light microscopy(H&E, PAS, Masson’s trichromeand PAS -Metheniminesilver stained slides)	• Diffuse, moderate tomarked, segmental to globalendocapillary hypercellularity(mostly neutrophilic)	• Focal, mild to moderate,segmentalendocapillaryhypercellularity(mostly mononuclear)	• Focal, absent to mild segmental endocapillary hypercellularity (mostly mononuclear)
	• ± Moderate tomarked mesangial hypercellularity	• ± Mild to markedmesangial hypercellularity	• ± Absent to moderate mesangial hypercellularity
Immunofluorescence	• Granular capillarywall (GBM), with ±mesangial IgG and/or C3deposits	• Variable granularmesangial C3± IgGwith ± capillary wall(GBM) deposits	• Variable granular mesangial C3± IgG with ± capillary wall (GBM) deposits
	• Mesangial IgA in IgA-dominant PIGN	• ± IgA, IgM, C1q,kappa or lambda	• ± IgA, IgM, C1q, kappa or lambda
	• ± IgA, IgM, C1q, Kappaor lambda		
Electron microscopy	• Numerous irregularly-spacedsubepithelial electrondense deposits, frequently inmesangial “notch”,rare “humps”, undergoingminimal to mild resorption	• Occasional to numeroussubepithelial electrondense deposits,a few “humps”, a few(at least 1) in mesangial“notch”, undergoingvariable (mild to marked)resorption	• Few subepithelial electron dense deposits, rare “humps” (up to 2), a few in mesangial “notch”, undergoing moderate to marked resorption
	• ±Rare intramembranous,mesangial, subendothelial	• ± Variable numbersof subendothelial,intramembranous, mesangial	• Numerous subendothelial, intramembranous, mesangial, undergoing moderate to marked resorption

Adapted from Haas, M., Hum Pathol, 2003. 34(1): p. 3–10.

### Statistical Analyses

Stata statistical software version 10 (Stata Corp, College Station, TX) was used for the statistical analysis. Continuous variables were reported as median and interquartile range (IQR). Continuous and categorical variables were compared using Wilcoxon rank-sum and the Fisher’s exact test, respectively. A *P*-value <0.05 was considered statistically significant. We used Kaplan-Meier plots and log-rank test to compare time to ESRD or death in groups with different kidney pathology features. Finally, we used Cox proportional hazards models to determine hazard ratios (HR) and 95% confidence intervals (CI).

## Results

### Pathological Findings

There were 1250 patients with a diagnosis of PIGN on renal biopsies performed from September 1998 to July 2013. After excluding 156 pediatric patients, 946 HIV-uninfected patients, 45 transplant recipients, 13 patients admitted at other institutions, there were 90 patients for evaluation. The blinded pathological review excluded 18 cases that were classified as immune-complex GN where there was no solid histologic evidence of PIGN. This resulted in 72 adult patients with confirmed PIGN and HIV-1 infection in the analysis (Figure 1). In the blinded pathological review of the archived renal tissues, 2 (3%) were designated as having acute PIGN, 42 (58%) persistent PIGN, and 28 (39%) healed PIGN. Three of the 72 PIGN cases (4%) were IgA-dominant PIGN. Twenty-six patients (36%) had PIGN as the sole diagnosis on their renal biopsy while 31 (43%) had PIGN with either concurrent classic FSGS or HIVAN and 15 (21%) have other pathological findings on renal biopsy.

### Clinical Characteristics of Participants with Different Histological Classification of PIGN

The majority of patients were African American (89%) and male in gender (64%). The median (interquartile range [IQR]) age at the time of the renal biopsy was 48 years (41, 53). The median (IQR) CD4 count and viral load at the time of biopsy were 322 cells/mm^3^ (156, 498) and 13,346 copies/mL (504, 100,566), respectively. The median (IQR) creatinine at the time of biopsy was 2.5 mg/dl (1.5, 4.9). Fifty-nine patients (82%) had hypertension and 17 (24%) had underlying diabetes mellitus. Forty out of 72 patients (56%) were coinfected with Hepatitis C. Only 12 patients (17%) exhibited a low C3 or C4. Bacterial infection was confirmed by positive blood culture in less than half of the patients (46%).

The two patients with acute PIGN were both African American males with HCV coinfection, hypertension, and history of intravenous drug use. Both presented with low C3 and normal C4 levels. At the time of renal biopsy, both were on antiretroviral therapy although one had a higher CD4 count and lower HIV-1 viral load (VL) compared to the other (CD4 657 vs. 50 and VL 400 vs. 939,000 respectively). However, the patient with better immunological status had underlying chronic kidney disease and ultimately required renal replacement therapy. The one with poor immune status had histological findings consistent with IgA-dominant PIGN. No preceding infection was identified in either patient.

The demographics and clinical characteristics of the remaining 70 patients with persistent and healed PIGN are summarized in Table 2. Hypertension and diabetes mellitus occurred in similar frequencies in the patients with persistent PIGN and healed PIGN (88% and 28% persistent PIGN vs. 79% and 23% healed, respectively). HCV coinfection was similar between the two groups, comprising 63% of patients with persistent PIGN and 56% of those with healed PIGN.

**Table 2:** Demographics, clinical characteristics and source of Infection of 71 patients at the time of diagnosis.

	PersistentPIGN (n = 42)	HealedPIGN (n = 28)	*P*-value
**Sex**			
Male (%)	27 (64)	17 (61)	0.8
Female (%)	15 (36)	11 (39)	
**Age (median, IQR)**	45 (41, 52)	48.5 (42, 54.5)	0.47
**Race - no. of patients (%)**			
Black	38 (91)	24 (86)	0.41
White	2 (5)	4 (14)	
Asian	1 (2)	0 (0.00)	
Others	1 (2)	0 (0.00)	
**Serum creatinine (Median, IQR)**	2.2 (1.1, 4.9)	2.6 (1.9, 5.1)	0.31
**CD4 count (Median, IQR)**	327 (199, 506)	238 (77, 459)	0.26
**Comorbidities - no. of patients (%)**			
Diabetes	11 (28)	6 (23)	0.8
Hypertension	35 (88)	22 (79)	0.34
Hepatitis C	24 (63)	14 (56)	0.61
Cirrhosis	1 (3)	2 (7)	0.57
Malignancy	8 (21)	3 (11)	0.34
Other systemic diseases	5 (13)	5 (20)	0.5
Intravenous drug use	19 (50)	10 (42)	0.61
**Low C3**	8 (25)	2 (10)	0.28
**Low C4**	8 (25)	2 (10)	0.28
**Site of Infection –no. of patients (%)**			
Endocardium	12 (29)	4 (14)	0.59
Bone/Joint	1 (2)	2 (7)	
Pleura	1 (2)	0 (0)	
Skin	4 (10)	4 (14)	
Lung/URTI	6 (14)	5 (18)	
Others	2 (5)	0 (0.00)	
None identified	16 (38)	14 (50)	
**Infectious Agent – no. of patients (%)**	N = 26	N = 25	
Staphylococcus	11 (42)	14 (56)	0.31
Streptococcus	7 (27)	8 (32)	
Others	8 (31)	3 (12)	
**HIV medications – no. of patients (%)**			
NRTI	21 (66)	11 (78)	0.5
NNRTI	7 (22)	2 (14)	0.7
PI	19 (59)	8 (57)	1
**Other medications – no. of patients (%)**			
ACE Inhibitors	19 (50)	12 (46)	0.8
ARB	1 (3)	1 (4)	1
Corticosteroid	9 (23)	3 (11)	0.51
Antibiotic use	24 (62)	16 (62)	0.92
**Nephrotic Proteinuria**	8 (26)	11 (46)	0.16
**Hematuria**	23 (62)	8 (30)	0.02

Most of the patients with persistent and healed PIGN were on antiretroviral therapy at the time of renal biopsy (66% persistent and 78% healed PIGN). The CD4 counts were also similar between the two groups. More patients with persistent PIGN (62%) exhibited statistically significant hematuria compared to those with healed PIGN (*P* = 0.02). In contrast, nephrotic-range proteinuria occurred more frequently in those with healed PIGN but these were not noted to be statistically significant (*P* = 0.16). The degree of renal dysfunction at the time of the renal biopsy was also similar between the 2 groups (median creatinine of 2.2 and 2.6 mg/dl). When identified, the heart or endocardium was the most common source of infection among patients with persistent PIGN while other infections were more frequently associated with healed PIGN (17%). However, the association between the source of infection and histopathological pattern was not statistically significant. In those with an isolated organism, *Staphylococcus* was identified as the most common infectious agent isolated in 42% of patients with persistent PIGN and 56% of those with healed PIGN.

Use of antimicrobials, angiotensin-converting enzyme inhibitor (ACE-I) and corticosteroid were similar between those with persistent PIGN and healed PIGN (*P = *0.92, *P* = 0.80 and *P* = 0.51, respectively).

### Pathological Findings

The two patients with acute PIGN have very similar pathological findings. Neither patient had crescents. Both of their renal biopsies showed subepithelial humps, subendothelial deposits and mesangial deposits. One of them had a positive C3 with trace IgM and intense IgA staining on IF while the other one was C3 negative. Both patients exhibited significant interstitial fibrosis, one with moderate interstitial fibrosis and the other with marked interstitial fibrosis. The pathological characteristics of the 70 HIV patients with persistent and healed PIGN are summarized in Table 3.

**Table 3:** Summary of pathological findings on 70 patients with persistent and healed PIGN.

Pathological Finding –no of patients (%)	Persistent PIGN (n = 42)	Healed (n = 28)	*p*- value
**Interstitial inflammation**			
Mild/Minimal	13 (31)	13 (45)	0.22
Moderate	14 (33)	8 (28)	0.80
Marked	5 (12)	3 (10)	1.00
No inflammation	10 (24)	4 (14)	0.38
**Crescents**			
Fibrocellular	1 (2)	0 (0)	1.00
Cellular	2 (5)	0 (0)	0.51
**Subepithelial humps**	31 (74)	17 (59)	0.30
**Subendothelial deposits**	24 (57)	2 (7)	<0.0001
**Mesangial deposits**	34 (81)	15 (52)	0.02
**Immunofluorescence**			
C3+ Ig	23 (55)	10 (34)	0.15
C3+ IgM	11 (26)	7 (24)	1.00
C3+ IgG	2 (5)	0 (0)	0.51
C3+ IgM+ IgG	10 (24)	3 (10)	0.22
C3 only	8 (19)	3 (10)	0.51
C3 negative	8 (19)	13 (45)	0.02
**Interstitial fibrosis**			
Mild	14 (33)	8 (28)	0.79
Moderate	11 (26)	9 (31)	0.60
Marked	7 (17)	5 (17)	1.00
No fibrosis	10 (24)	7 (24)	1.00

Only 3 patients out of 70 were found to have crescents on renal biopsy, all of whom had persistent PIGN. One had a fibrocellular crescent while the two others had cellular crescents. IF was positive for C3 and Ig (either IgA, IgM or IgG) on 55% of patients with persistent PIGN and 36% of patients with healed PIGN. Among the patients with positive C3 and Ig on IF, majority of the patients, both in the persistent and healed PIGN, had IgM positive deposits along with C3 (26% persistent PIGN group and 25% healed PIGN group). A small proportion of patients on both groups (19% persistent PIGN and 11% with healed PIGN) had IF that was positive for C3 only. Subepithelial humps were identified in in the majority of patients with persistent and healed PIGN. In contrast, subendothelial deposits were significantly predominant in patients with persistent PIGN compared to those with healed PIGN (57% vs 7%, *P* = <0.0001). The majority of the patients with persistent PIGN (76%) had interstitial fibrosis, mostly mild and moderate interstitial fibrosis (33% and 26% respectively). The same percentage of patients with healed PIGN (76%), had interstitial fibrosis which were mostly moderate (31%) and mild (28%). Similarly, interstitial inflammation was noted in 76% of patients with persistent PIGN and 86% of patients with healed PIGN.

### Mortality and Incidence of CKD

The patients were followed up for a median (IQR) of 17 months (1–45 months). Mortality occurred in 14 out of 72 patients (19%) during the follow-up period. Among those who died, 6 patients had a sole diagnosis of PIGN, 5 with PIGN and other pathologies and 3 have PIGN with FSGS or HIVAN. Two of the patients with PIGN who died exhibited histopathological features of IgA-dominant PIGN.

Fourteen patients (19%), including one with acute PIGN, required renal replacement. None of the patients with IgA dominant PIGN required renal replacement therapy. We found no significant association between the histopathological classification of PIGN and ESRD. The cumulative progression to ESRD was similar in patients with persistent PIGN and healed PIGN (19% and 18%, respectively; *P* = 0.95 log-rank test). Individuals with healed PIGN had a similar risk of progressing to ESRD as those with persistent PIGN (HR, 0.96; 95% CI, 0.31 to 3.00). Similarly, the cumulative incidence of ESRD in patients with an exclusive diagnosis of PIGN was similar to patients with PIGN in combination with HIVAN or FSGS, and PIGN with other pathologies (15%, 25% and 14%, respectively; *P* = 0.72 log-rank test) (Table 4). Compared to those with a sole diagnosis of PIGN, the risk of progressing to ESRD was similar in those with PIGN plus HIVAN or FSGS (1.09; 95% CI 0.28–4.18) and those with PIGN plus other pathologies (0.66; 95% CI 0.11–4.06).

**Table 4:** Estimated rates of mortality and ESRD 12 months following kidney biopsy, according to pathologic findings.

No. of patients (%)	PIGN only (n = 26)	PIGN+FSGS/HIVAN (n = 31)	PIGN + other* (n = 15)	*P*-value**
Mortality	6 (23)	3 (9)	5 (36)	0.09
ESRD	4 (15)	8 (25)	2 (14)	0.74

*Others: MPGN, lupus-like, membranous, diabetic glomerulosclerosis.

**Log-rank test.

**Figure 2:**
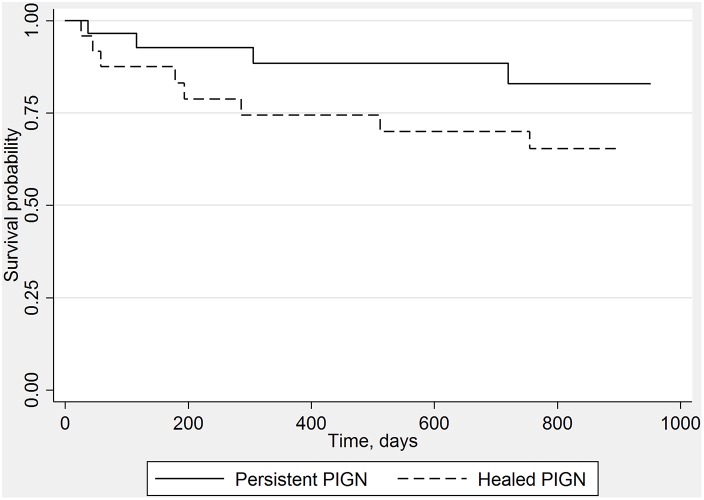
Mortality according to histopathological pattern of PIGN: Patients with healed PIGN had significantly worse survival compared to those of persistent PIGN, log-rank test.

Mortality rates, on the other hand, showed differences based on the histopathological pattern of PIGN. The cumulative incidence of mortality in individuals with healed PIGN was higher compared to those with persistent PIGN (36% and 10%; *P = 0.05 log-rank test*) (Figure 2). This significant difference was eliminated in a separate analysis where cases with co-existing histopathological diagnoses were excluded. Including only those with pure PIGN, there were 15 patients with persistent PIGN and 10 patients with healed PIGN. Of these, 2 and 4 patients died respectively and the log-rank P value for this comparison was 0.41 (data not shown). However, the significant drop in the sample size may limit the interpretation of this result. Patients with IgA-dominant PIGN did not show increased mortality (*P = 0.69*) or increased need for renal replacement (*P* = 0.16).

## Discussion

This is the first study to explore the clinical and pathological characteristics of PIGN in HIV- infected individuals. In this study *Staphylococcus* was the most common etiology of an identified infection prior to diagnosis of PIGN. Interestingly, renal outcome was not influenced by the histopathological pattern, but mortality was relatively high (19%) in this cohort during the follow-up period, with patients with healed PIGN having greater mortality.

PIGN has been traditionally linked to Streptococcal pharyngitis or skin infection [13]. In our HIV-infected cohort with PIGN, *Staphylococcus* was the most common causative agent identified. This supports the findings of various authors, who described a shift in the epidemiologic features of PIGN over the past decades [8], [9]. Nasr *et al* described *Staphylococcus* as the most common causative agent in the elderly population [16] and in IgA dominant PIGN [17]. Our study showed that this is true even in younger patients with HIV-1 infection with no pathological features of IgA-dominant PIGN.

In our HIV-infected cohort, bacterial infection was only confirmed in 46% of the patients, which is consistent with a study which revealed that in as much as 24% to 59% of patients with PIGN, the causative organism cannot be identified [13]. The low yield of bacterial culture in identifying the causative organism could be due to the diversity of organisms that can cause PIGN which include viruses and parasites. Furthermore, the role of HIV and HCV infection in the development of PIGN in this cohort is not clear although one can argue that antigens of these viruses may be playing a role in this process. Hepatitis C infection was noted in more than 50% of our HIV-infected cohort. However, the presence of HCV coinfection did not increase predisposition for a specific histological pattern of PIGN. Previous studies reported that HCV coinfection with HIV-1 increases predisposition to immune-complex glomerulonephritis [18]. HIV-1 itself may promote an increase in circulating immune complexes by promoting polyclonal hypergammaglobulinemia [19]. One study also suggested that formation of anti-HIV antibodies might play a role in forming immune complexes in some HIV patients [20] Whether HIVICK is caused by passive trapping of the circulating immune complex, or in situ deposition of antibody-antigen complex, is still not completely understood.

The lack of clinical signs of infection may explain the lower frequency of acute pattern of PIGN in our cohort. The majority of our patients with PIGN had healed and persistent PIGN on renal biopsy, and only 2 had acute PIGN. One study reported that as much as 16% of adult patients may not have any clinical manifestation of infection [13]. The absence of overt signs of acute infection is believed to be one of the reasons for delayed diagnosis [10]. Although, the patients with PIGN had comparable degrees of renal dysfunction regardless of the histopathological pattern, early diagnosis in the HIV population may be important since patients with healed PIGN tend to have a higher mortality. Hence, PIGN should always be entertained as a differential diagnosis in HIV patients with renal dysfunction, even in the absence of a source of infection. Given the heterogeneity of renal pathologies in HIV as well as the absence of a reliable clinical indicator of PIGN in HIV patients, a timely renal biopsy is very valuable in this population.

Our study comprising of predominantly male African American HIV-infected patients, a large proportion (82%) of the patients were hypertensive. This is slightly higher than what has been previously reported [13]. However, the occurrence of hypertension did not differ between the patients with different histologic pattern of PIGN. Conversely, diabetes mellitus, which has been identified as the most frequent risk factor in a study of adults with acute PIGN [13], was only documented in 17 patients (24%) in our HIV-infected cohort. This finding may be partially explained by the demographic differences with other studies and suggests that diabetes mellitus is not a major risk factor for PIGN among young African American patients with HIV-infected patients.

Our study showed that individuals with persistent PIGN had a similar risk of progressing to ESRD as those with healed PIGN. Two prior PIGN studies identified the absence of interstitial inflammation as one of the pathological findings which correlates with renal recovery [11], [13]. Interstitial fibrosis was identified by *Montseny* et al as one of the predictors of persistent renal dysfunction [8]. In our cohort of patients, the proportion of patients with interstitial inflammation and interstitial fibrosis were very similar between the persistent PIGN and healed PIGN groups. Perhaps, this explains why the mean creatinine at the time of renal biopsy and progression to ESRD during the follow-up period were similar between the two groups were similar. The association between the presence of crescents and renal outcome seems to be less straightforward in PIGN. While *Nasr* et al [13] did not find a correlation between crescents and renal outcome, another study reported poorer renal outcomes with crescentic PIGN [21]. In our patient population, only 3 patients exhibited crescents on renal biopsy. Owing to the low number of patients with crescentic GN, we cannot determine if there is a correlation between renal outcome and crescentic glomerulonephritis in HIV-infected patients. It might be worthwhile to look into this on future studies involving a larger number of HIV patients with PIGN.

Studies on HIV-infected population have suggested that HIVAN progresses more rapidly than non-HIVAN renal diseases [1], [22], [23]. In our study, some of the patients with PIGN had concomitant findings consistent with HIVAN. However, these patients did not show an increased progression to ESRD compared to those patients with the sole diagnosis of PIGN or with PIGN in conjunction with other pathologies. The short follow-up period of our study may not have limited our ability to distinguish differences. Perhaps and more importantly, a substantial proportion of our patients were on antiretroviral therapy at the time of diagnosis, which could have delayed the progression of HIVAN in this cohort to ESRD.

Although renal outcome did not differ between the persistent and healed PIGN groups, healed PIGN appears to correlate with higher mortality during the follow-up period. Although this may be counterintuitive and there is real possibility of a chance finding, there is evidence that at the time of biopsy more patients with healed PIGN exhibiting more nephrotic-range proteinuria which may have been associated with worse outcome in this group of patients. Alternatively, since we did not account for the timing of institution of antibiotic and HAART use, this could have played a crucial role in the different mortality between the 2 groups.

Our study has several limitations. It is a single-center study with patients mostly coming from an inner city urban hospital. Our patient population consisted mostly of African American patients; hence the results may not apply to other populations. Only patients who had renal biopsies were included, which potentially excluded the HIV patients who are coagulopathic or with severe illness. We also did not account for the duration of HIV-1 infection or timing of initiation of HAART or antibiotic therapy, which could have an impact on the renal outcome or mortality. Although the histological pattern proposed by Haas is not widely utilized in practice, our study was an attempt at using some form of standardization for the study.

In conclusion, PIGN is a common histopathologic diagnosis in HIV patients. *Staphylococcus* is the most commonly identified organism in a preceding infection in HIV-associated PIGN. The histopathological classification of PIGN has no bearing on the renal outcome but this group of patients incur overall high mortality rate. Patients with healed PIGN have the highest mortality suggesting that delayed diagnosis may have a negative impact on overall outcome.
